# A novel gene *OsAHL1* improves both drought avoidance and drought tolerance in rice

**DOI:** 10.1038/srep30264

**Published:** 2016-07-25

**Authors:** Liguo Zhou, Zaochang Liu, Yunhua Liu, Deyan Kong, Tianfei Li, Shunwu Yu, Hanwei Mei, Xiaoyan Xu, Hongyan Liu, Liang Chen, Lijun Luo

**Affiliations:** 1 Shanghai Agrobiological Gene Center, Shanghai Academy of Agricultural Sciences, Shanghai 201106, China; 2 National Key Laboratory of Crop Genetic Improvement, College of Plant Science, Huazhong Agricultural University, Wuhan 430070, China

## Abstract

A novel gene, *OsAHL1,* containing an AT-hook motif and a PPC domain was identified through genome-wide profiling and analysis of mRNAs by comparing the microarray of drought-challenged versus normally watered rice. The results indicated *OsAHL1* has both drought avoidance and drought tolerance that could greatly improve drought resistance of the rice plant. Overexpression of *OsAHL1* enhanced multiple stress tolerances in rice plants during both seedling and panicle development stages. Functional studies revealed that *OsAHL1* regulates root development under drought condition to enhance drought avoidance, participates in oxidative stress response and also regulates the content of chlorophyll in rice leaves. *OsAHL1* specifically binds to the A/T rich sequence region of promoters or introns, and hence directly regulates the expression of many stress related downstream genes.

Water shortage is continually a limiting factor in crop production, which in turn imposes a negative impact on the food security of the world. For this reason, scientists have never stopped efforts to improve efficiency of water usage and reduce the affect of water shortages on crop production. There has been a particular focus on improving rice production by creating drought resistant genotypes. To this end many drought stress response or drought tolerance related genes have been uncovered^1^,^2^,^3^,^4^,^5^,^6^,^7^. However, functional studies on the role of these drought response and tolerance related genes lags behind the discoveries of the genes themselves and currently known genes do not provide a practical solution to the draught problem^8^,^9^.

Why have drought stress response/drought tolerance related genes not solved the problem? Drought resistance is a complicated trait that involves a network of many gene modules. There isn’t a single, magical drought-tolerance gene, because instead plants have evolved complex mechanisms to deal with water shortages^9^. Drought resistance is divided into four components: drought escape, drought avoidance, drought tolerance, and drought recovery^8^. If the four components could be combined and introduced into rice plant as a single trait, then drought resistance could be improved greatly. But it is often difficult to combine several components into one trait. Yue *et al.* reported the genetics of drought avoidance and drought tolerance are separated^10^. Here we reported that a novel gene *OsAHL1*, containing an AT-hook motif and DUF296 domain, provides both drought avoidance and drought tolerance. Therefore, this gene could greatly improve drought resistance of rice plants.

## Results

### *OsAHL1* identified through analysis of drought related QTLs and gene microarray

In our previous study using a RIL population from the cross between Zhenshan97B (an *indica* lowland rice) and IRAT109 (a *japonica* upland rice), two drought avoidance linkage QTLs were located at the same site in Chr11, which were related to the deep root rate of rice under normal irrigation/drought stress (RM286-RM332)^11^. Meanwhile, the result of gene expression through microarrays showed that a novel gene (LOC_Os11g05160) was significantly up-regulated by drought stress on this QTL (the data was unpublished). As a result, this novel gene was cloned from the cDNA of IRAT109 by direct PCR, and was nominated as *OsAHL1* (AT-hook content nuclear localized protein).

### *OsAHL1*, responsive to multiple stresses, is induced by drought stress in vascular bundles

**Figure 1:**
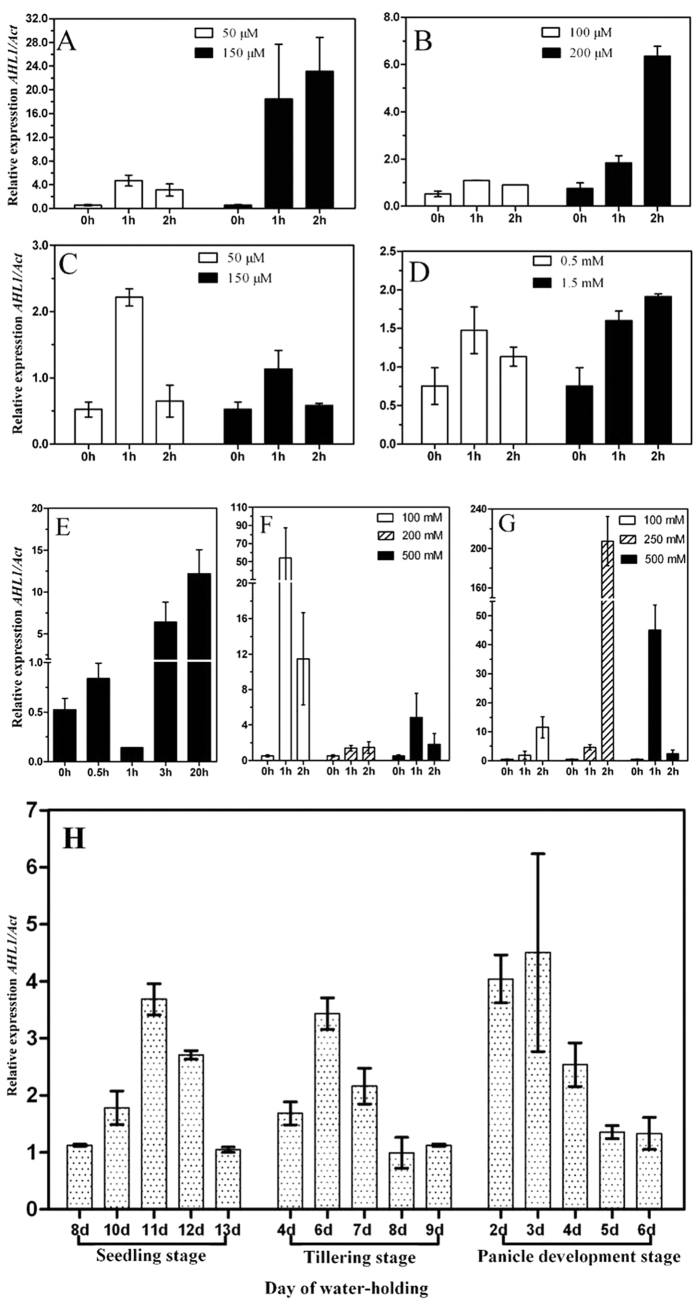
Relative expression of *OsIAHL1* in rice. The relative expression of *OsAHL1*gene in cultivar IRAT109 (*Oryza sativa* L. *ssp japonica*) with the plant hormones (**A–D**), and dehydration (**E–H**). *OsAHL1*gene responded to plant hormones (**A**) ABA, (**B**) H_2_O_2_, (**C**) JA, (**D**) SA, and dehydration, such as (**E**) cold (4 °C), (**F**) Mannitol, (**G**) NaCl treatment in 4 leaf stage; and (**H**) under water withholding condition in different developmental stages. The plants grown in normal watering conditions were used as a control. Error bars are the SD based on 3 replicates.

Real time qPCR has been used to study *OsAHL1* expression pattern in the seedlings of rice plant cultivar IRAT109 grown under different conditions. The result showed that, *OsAHL1* was up-regulated by plant-hormones, such as ABA, H_2_O_2_, JA and SA. Each of these hormones elicited a dosage and time dependent effect on *OsAHL1* (Fig. 1A–D). Similarly, abiotic stress provoked by cold (Fig. 1E), Mannitol (Fig. 1F), and NaCl (Fig. 1G) also induced an up-regulation in *OsAHL1* gene expression. Analysis of relative expression level was carried out at the 4 leaf stage, tillering stage, and panicle development stage under natural water-holding condition. The results indicated that during each of the three stages *OsAHL1* was continuously up- regulated at the beginning and subsequently returned to normal levels (Fig. 1H).

**Figure 2:**
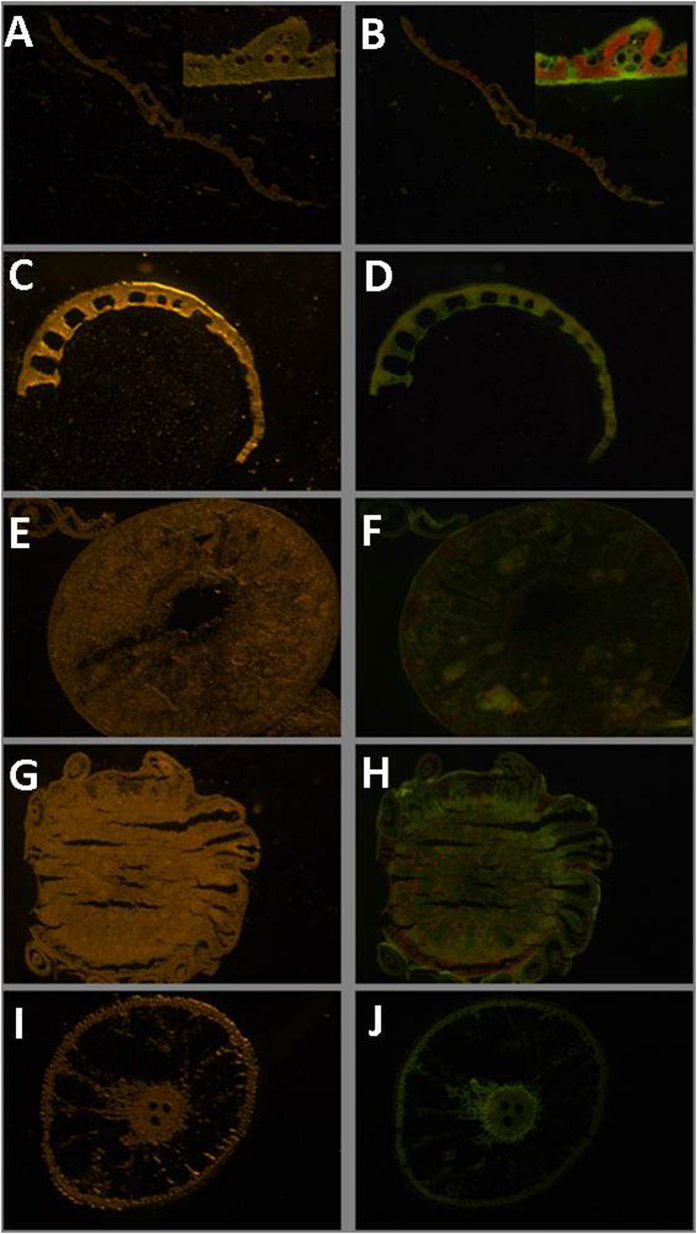
Expression pattern of GFP driven by the *OsAHL1* promoter (P_AHL1_) in transgenic rice plants under normal conditions. Panels on the left were taken under visible light and the right under UV. GFP signal was monitored in vascular bundles of leaf blade (**A,B**), leaf sheath (**C,D**), stem internode (**E,F**), stem node (**G,H**) and root (**I,J**).

To examine the expression profiles of *OsAHL1*, transgenic rice plants containing the P_*AHL1*_::GFP (green fluorescent protein gene under control of the 1.7 Kb *OsAHL1* promoter) construct were generated and the GFP expression pattern was monitored under normal conditions (Fig. 2). The GFP signal was observed under UV spectrum in all of the root systems. Additionally, the GFP signal was monitored in vascular bundles of leaf sheath, blade, and stem (node and internodes).

### Overexpression of *OsAHL1* enhanced drought, salt and cold stress tolerance in rice seedling stage

To test the function of *OsAHL1*in rice, the full-length cDNA of *OsAHL1* driven by CaMV35’s promoter was transformed into rice cv. Zhonghua 11; 25 independent transgenic plants were obtained and confirmed by PCR and Southern blot analysis. Three independent transgenic lines with overexpression of *OsAHL1*were selected to examine their performance on multiple stress tolerance tests. For testing the abiotic stress tolerance of transgenic plants we used three different methods to simulate abiotic stress at the 4 leaf stage of rice seedlings. Over 30 transgenic plants from each transgenic line were examined in each test.

**Figure 3:**
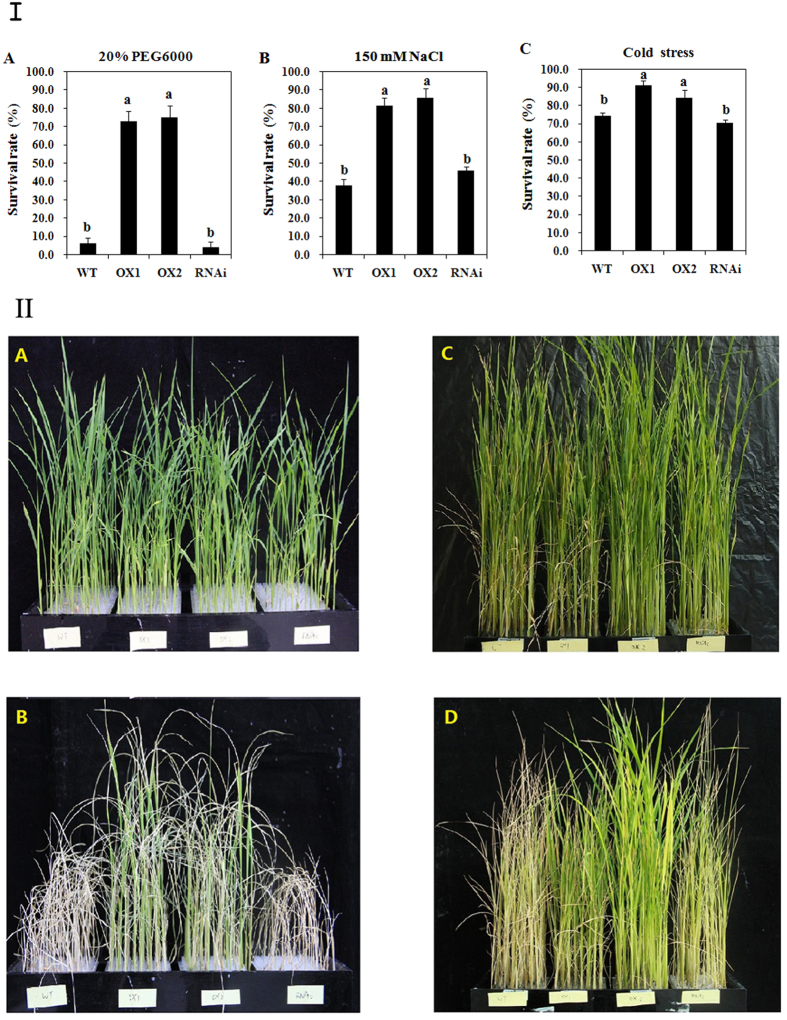
Overexpression of *OsAHL1* gene enhanced abiotic stress resistance of rice at the seedling stage. In panel I, the survival rate of transgenic (I17 lines), WT and RNAi plants at the seedling stage was investigated under 20% PEG(I-A), 150 mM salt (I-B) and 4 °C (I-C) stresses. Figure panel II is the growth status of *OsAHL1* transgenic lines, WT and RNAi plants. II-A and II-B were before and at the end of seedling test in 20% PEG600 respectively, while II-C and II-D were in 150 mM salt stress. The 4 leaf rice plants were transplanted in hydroponic culture medium and treated with the above stresses for 7 days, then returned to normal condition for another 7 days. Error bars are the SD based on 3 replicates; every replicate contains 48 individual plants. Data bars in panel I with no superscripts in common indicate significant difference at *p* < 0.01.

Rice seedlings at the 4 leaf stage were grown in 20% PEG6000 (simulating drought stress) for 4 days and then returned to normal condition for another 7 days. Result showed that more than 70% of transgenic line plants recovered after osmotic stress, while almost no plants recovered in wild type (WT) and RNAi plants (Fig. 3I-A, II-A,B). All of the transgenic lines over-expressing *OsAHL1* showed an increased survival rate.

Salt tolerance tests produced a similar trend as that seen during the PEG osmotic stress. After treatment with 150 mM NaCl for 3 days, almost all of the WT plants were wilted while the *OsAHL1* over-expressed plants survived the salt stress without serious rolling leaves. Followed by 7 days of normal watering more than 82% of *OsAHL1* over-expressed plants survived the salt stress, while less than 40% of the WT plants survived the salt stress (Fig. 3I-B, II-C,D).

Cold tolerance of the transgenic lines was also tested. Rice plants were grown in liquid medium until the 4 leaf stage, transferred to a growth chamber and cooled down to 5 °C. After 7 days of growth in a 5 °C chamber, the temperature was raised to 25 °C for 7 days. At the conclusion of the 14 days the survival rate of seedlings was calculated. When compared with the WT and RNAi plants, survival rate of *OsAHL1* over-expressed plants increased more than 10 percent (Fig. 3I-C). Overexpression of *OsAHL1* in the seedling stage of rice demonstrated increased tolerance to abiotic stresses, especially to stress caused by water deficit.

### Overexpression of *OsAHL1* also significantly improved drought resistance at the panicle development stage

**Figure 4:**
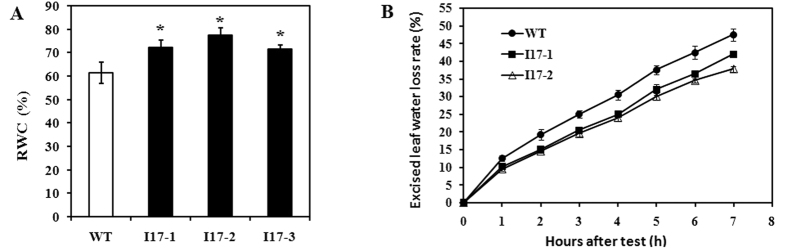
Over-expression of *OsAHL1* gene improved water-content ability of rice leaves. (**A**) The relative water content (RWC) of leaves under drought stress. (**B**) The rate of water loss for separated leaves. Error bars are the SD based on 3 replicates. *Indicates significant difference by the t-test at *p* < 0.05, compared to WT.

Rice plants were grown in polyvinyl chloride (PVC) pipes. Mild drought stress was generated from the early panicle development stage, according to a defined protocol^11^. During the process of gradual drought stress, water loss from the leaves of *OsAHL1* over-expressed plants was much slower. Therefore, the relative water content in the leaves of *OsAHL1* over-expressed plants was higher than that in the WT, causing delayed leaf-rolling during the mild drought stress (Fig. 4).

**Figure 5:**
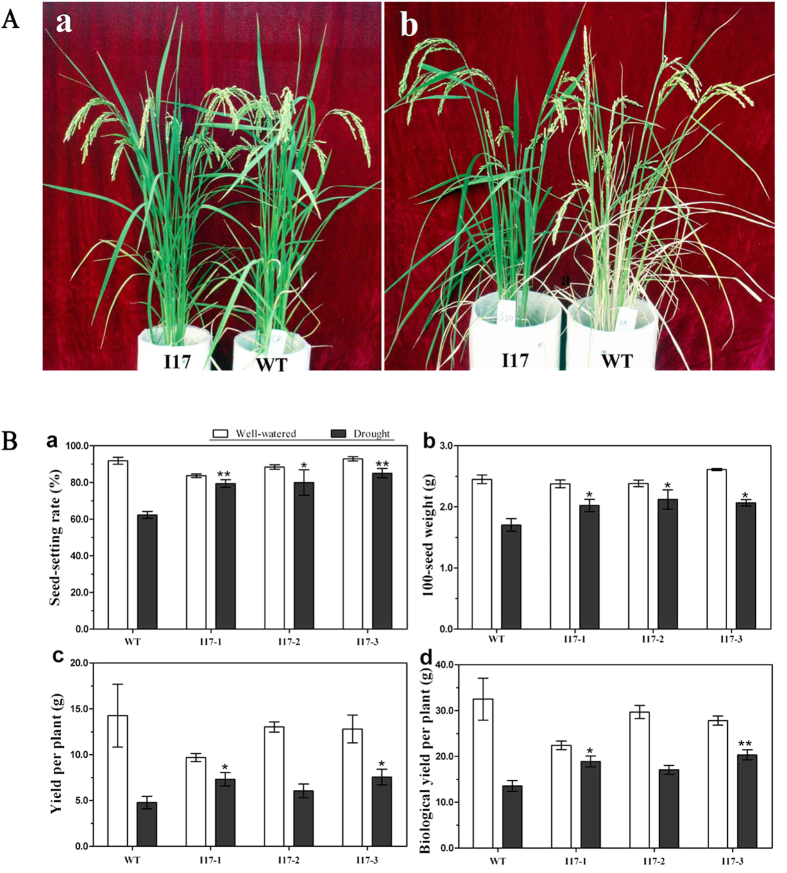
Overexpression of *OsAHL1* improved drought resistance at reproductive stage. (**A**) *OsAHL1 overexpressed* plants (I17 lines) and WT under well-watered conditions (a), and drought stress (b). (**B**) Agronomic traits under normal conditions and drought stress: (a) seed-setting rate, (b) grain yield per plant, (c) the biological yield per plant, and (d) the 100-seed grain weight. Error bars are the SD based on 4 (welled-water) or 8 (under drought) plant replicates. *Indicates significant difference by the t-test at *p* < 0.05, compared to WT; **Means significant difference at *p* < 0.01.

Agronomic traits were investigated after harvesting. Result showed that all of *OsAHL1* over-expressed plants exhibited higher spikelet fertility (17~23% higher) (Fig. 5Ba), yield per plant (more than 27%) (Fig. 5Bc), biomass yield (25~50% higher) (Fig. 5Bd) and higher 100-seed weight (more than 29%) (Fig. 5Bb) when compared to the WT. Meanwhile, under well irrigated conditions, all *OsAHL1* over-expressed plants and WT plants showed similar performance for these agronomic traits. These results display that over-expression of *OsAHL1*did not affect growth and yield productivity of rice grown under normal conditions, but increased the ability degree of drought tolerance under water-limited conditions.

### *OsAHL1* was involved in some oxidative stress response and regulated the content of chlorophyll in rice leaves

**Figure 6:**
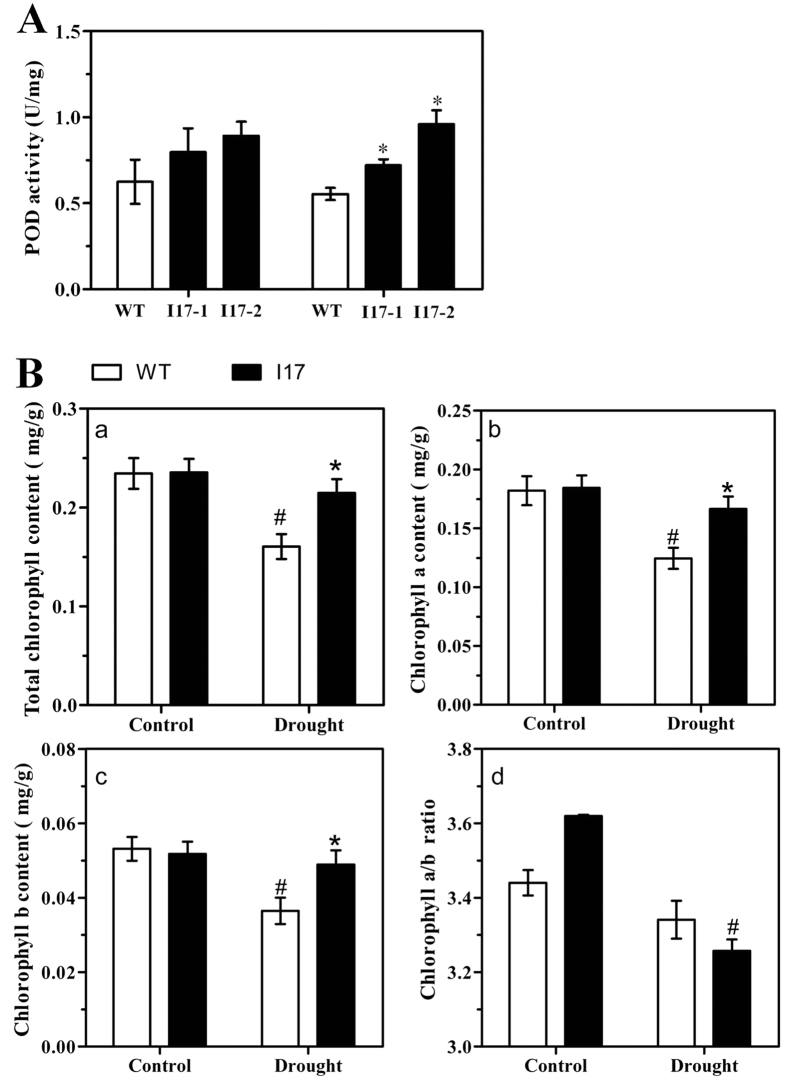
*OsAHL1*-Overexpression improved POD activity and regulated chlorophyll content in the leaves of rice. (**A**) POD activity of I17 plant lines and WT under normal and drought conditions. (**B**) Comparison of leaf chlorophyll content (a), chlorophyll composition (b,c) and chlorophyll a/b ratio (d) between I17 plant lines and WT under normal and drought condition. Error bars are the SD based on 3 replicates. *Indicates significant difference by the *t*-test at *p* < 0.05, compared to WT plants under the same condition; ^#^Means significant difference by the *t*-test at *p* < 0.05, comparison between control and drought stress for the same plant line.

Because overexpression of *OsAHL1* resulted in improved drought resistance in rice plants, the function of *OsAHL1* in oxidative stress response was further investigated further. The activity of the ROS scavenging enzyme, peroxidase (POD), was measured in *OsAHL1*-overexpressing lines. The result showed that the *OsAHL1*-overexpressing plants had a significantly higher activity of POD than the WT plants (Fig. 6A). It suggests that *OsAHL1* helps to remove the peroxidase, increasing the stabilization of plasma lemma under drought stress.

Chlorophyll is an important biochemical trait marker of drought tolerance in plants. Compared with the contents of total Chlorophyll and Chlorophyll a, Chlorophyll b in the leaves of plants in the control (under normal watering condition), plants grown under drought stress had a decreased content of all these Chlorophyll components. But, *OsAHL1*-overexpressing plants showed significantly less decreased than the WT plants (Fig. 6B). Chlorophyll a/b ratio in the leaves of *OsAHL1* plants increased under normal condition, but decreased under drought stress. The above results suggest that *OsAHL1* leads to decreased degradation of chlorophyll and therefore modulates the ratio of chlorophyll a/b, thus helping rice adapt to drought stress.

### *OsAHL1* regulated root development under drought condition to enhance drought avoidance

**Figure 7:**
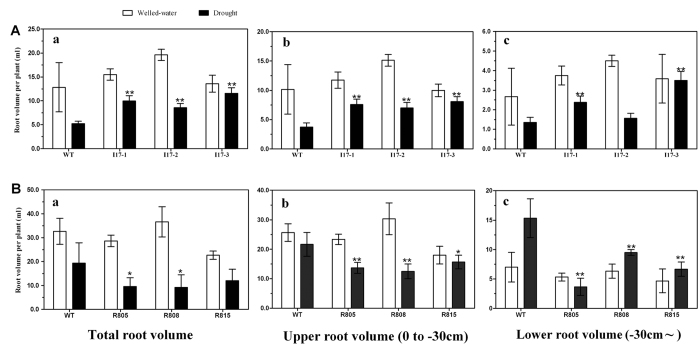
*OsAHL1* regulated the development of rice root system under drought conditions at the reproductive stage. Root volume per plant between *OsAHL1* overexpression (I17) plant lines (**A**) and *OsAHL1*-RNAi plant lines (**B**) compared with WT under normal and drought conditions. (a) Total root volume, (b) upper root volume, and (c) lower root volume. Error bars are the SD based on 3 replicates. *Indicates significant difference by the t-test at *p* < 0.05, compared to WT; **Means significant difference at *p* < 0.01.

Because the *OsAHL1* gene was located in the QTL region associated with root development in rice under control/drought stress, root traits of *OsAHL1* transgenic and WT plants were investigated (Fig. 7A). Results revealed that under normal watering conditions there was no significant difference in root volume between *OsAHL1* over-expressed and WT plants. But under drought stress conditions root volume of both *OsAHL1* over-expressed and WT plants was decreased, with a larger decreased seen in WT plants. In other words, total root volume, upper root volume and lower root volume in a plant were much higher in *OsAHL1* over-expressed plants than in WT plants under drought stress conditions.

For further confirmation, RNAi lines of the *OsAHL1* gene were created by the amiRNA method, introducing a dehydration response promoter of *OsHVA22*^12^ to down regulate *OsAHL1* expression in rice plants (Fig. 7B). The difference in root traits between RNAi plants of the *OsAHL1* gene and WT plants was similar to the previously described results. Under normal watering conditions RNAi plants of the *OsAHL1* gene and WT plants showed similar total root volume, upper root volume and lower root volume. However, under drought stress RNAi plants of the *OsAHL1* gene were shown to display significantly reduced root volume when compared to the WT plants.

Accordingly, compared with WT plants, overexpression of *OsAHL1* in rice was beneficial to drought response and promoted root development while down-regulation of *OsAHL1* resulted in an opposing effect by diminishing the root system growth and development. As we know, the root system is the most important organ for water absorption, here our results uncovered that the *OsAHL1* gene could enhance drought avoidance in rice, via regulation of root development.

### OsAHL1 protein contains an AT-hook and a PPC domain, both of which could guide nuclear localization of protein

The *OsAHL1* gene encodes an uncharacterized protein containing 366 amino acids with an AT-hook, and a Domain of Unknown Function 296 (DUF296) which was named the PPC domain (Plants and Prokaryotes Conserved domain)^13^.

**Figure 8:**
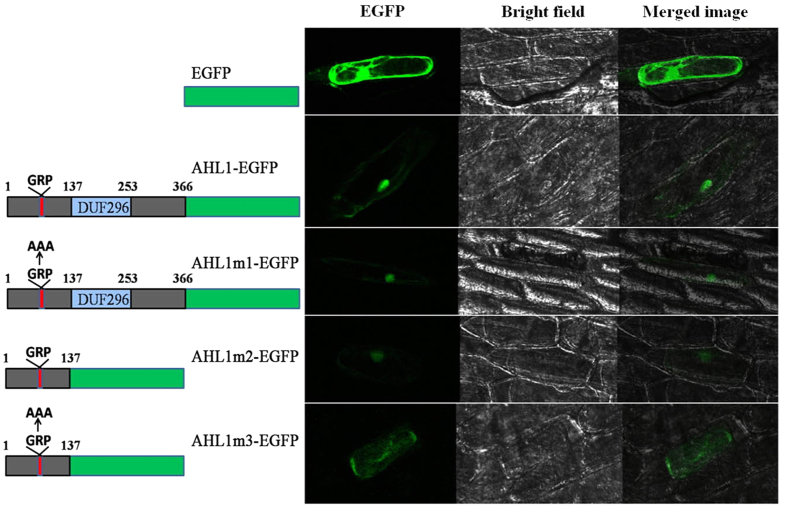
The role of AT-hook and DUF296 on nuclear localization of OsAHL1. The figure panels are confocal microscopic images of OsAHL1-EGFP fusion protein driven by CaMV35s promoter in onion epidermal cell. The EGFP driven by CaMV35s promoter was used as positive control. The left schematic diagrams represent the expressed proteins. The AT-hook motif was indicated by red line, and DUF296 was indicated by blue box.

To test the functions of the AT-hook and PPC domain we constructed a series of expressing vectors for plants that encode either a full-length or one of three types of mutant *OsAHL1* protein, each with green fluorescent protein (EGFP) fused to the C terminus (Fig. 8). The *Mutant 1* protein had the 3 core amino acids of the AT-hook replaced by alanine (GRP to AAA); *Mutant 2* protein had the DUF296 domain and all domains after it deleted; *Mutant 3* was an expression vector based on *mutant 2* but also the 3 core amino acids of the AT-hook were replaced by alanine (GRP to AAA). After construction of these modified expression vectors they were introduced into onion epidermal cells by the microprojectile bombardment method and the transient expression of these fused GFP proteins were examined.

The results showed that the full-length OsAHL1 protein (AHL1-GFP), mutant 1 (AHL1m1-GFP) and mutant 2 (AHL1m2-GFP) proteins were localized in the cell nucleus; however, the mutant 3 (AHL1m3-GFP) protein was found spread throughout the cell (Fig. 8). It was confirmed that two regions, the core AT-hook and DUF296 domain and/or the entire region after the DUF296 domain in the *OsAHL1* gene sequence, could guide AHL1 proteins to the nucleus.

We used the Yeast-Two-Hybrid system to test the homologous binding capacity of these functional domains. The results showed that as long as the protein contains the complete PPC domain binding will occur. It follows that this interaction is maintained by the PPC domain in each subunit. The results are similar to Lin’s study, where the PPC containing protein could form a trimer^14^.

**Figure 9:**
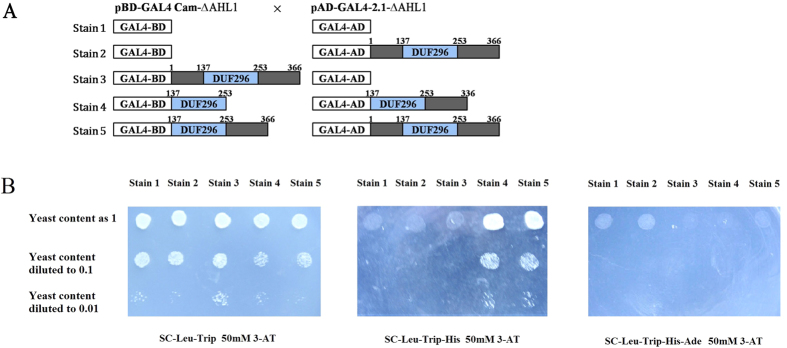
Yeast Two-hybrid tested the homologous combination capability among OsAHL1 proteins (or its DUF296 domains). (**A**) Vector construct for yeast expression of OsAHL1 protein. (**B**) OsAHL1 proteins or its DUF296 domains could be bound together in yeast stain YRG-2. Yeast were diluted and dotted on amino acid deficiency SD medium with 50 mM 3-AT.

More importantly, taking look at stain 3 together with stain 1 and stain 2 on SD-Leu-Trip-His +50 mM 3-AT selection medium, no self-activation of OsAHL1 proteins was detected in yeast (Fig. 9). To the opposite, stain 4 and stain 5 which contain the complete PPC domain or the complete OsAHL1 protein, reporting gene was activated on SD-Leu-Trip-His +50 mM 3-AT selection medium, but no activation was observed on SD-Leu-Trip-His-Ade +50 mM 3-AT selection medium, indicating there is no self-activation of BD-AHL1 and AD-AHL1. The results indicate that OsAHL1 and its DUF196 domain have the homologous binding ability.

### OsAHL1 protein regulates the expression of many stress related genes directly

**Figure 10:**
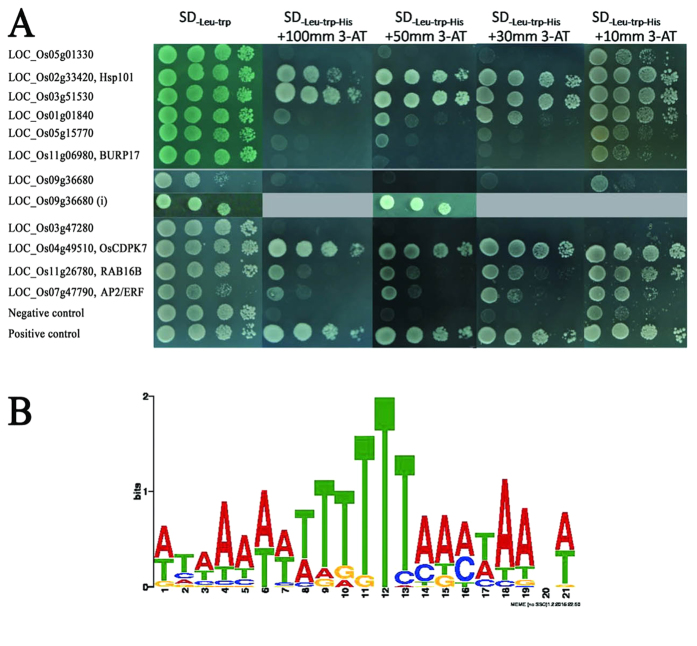
OsAHL1 binds to the target genes directly. Yeast One-Hybrid Assay was used to analyze the interactions between OsAHL1 protein and the DNA region of its candidate target genes. Yeast were diluted and dripped on amino acid deficiency SD medium with gradient 3-AT. The genes were labeled on the left, and “i” indicates the intron region, the others are the promoter region of the genes. (**B**) The consensus of DNA motifs that OsAHL1 protein interacts with are predicted by MEME.

**Figure 11:**
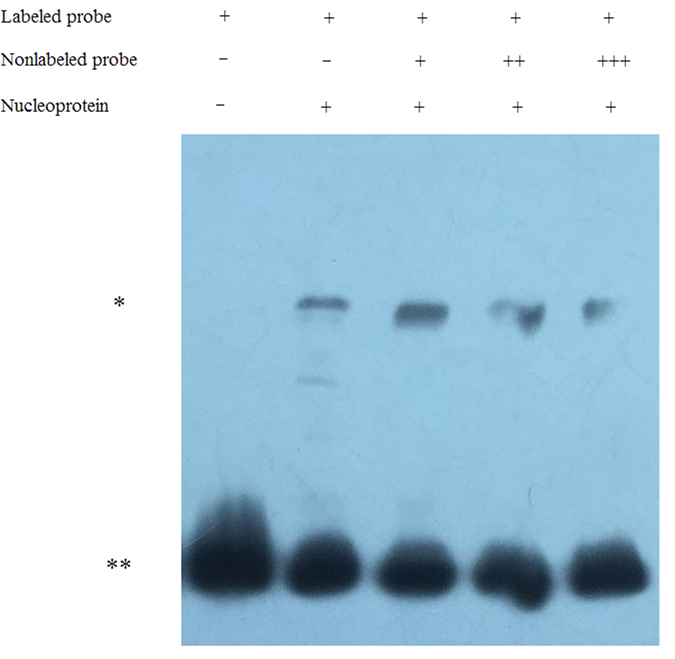
EMSA results. Electrophoretic mobility shift assay (EMSA) using oligonucleotide probes containing the OsAHL1 binding region. The **denotes unbound biotin labeled DNA, *denotes OsAHL1-DNA (gene LOC_Os02g33420) complex.

We used the Affymetrix transcriptome microarray to analyze the expression patterns of the *OsAHL1* overexpression rice plants, WT and the RNAi plant lines. As a result 14 differential genes were identified through comparison of the expression profiles of *OsAHL1* overexpressed, WT and RNAi plant lines. The promoter regions of these differential genes were cloned by PCR using the sequence specific primers, which were used for yeast one hybrid analysis (Y1H) to detect the interaction of these promoters with OsAHL proteins. Results showed OsAHL1 protein could bind directly to the promoter regions of the following 7 genes: LOC_Os05g01330, LOC_Os02g33420 (HSP101), LOC_Os03g51530, LOC_Os01g01840 (bHLH like), LOC_Os04g49510 (OsCDPK7), LOC_Os11g26780 (dehydrin Rab16b), LOC_Os07g47790 (AP2-ERFBP like). OsAHL1 protein could bind to the intron region of *LOC_Os09g36680* (*OsRNS4*) but did not bind to its promoter region (Fig. 10A). We noticed that some of the interactions showed in Y1H results appear to be relatively weak, so electrophoretic mobility shift assay (EMSA) was conducted to further confirm the interaction. EMSA result indicated that OsAHL1-DNA complex, the product that OsAHL protein interacts with the DNA sequence in the promoter region of the target gene LOC_Os02g33420 (HSP101), was detected (Fig. 11), which further explains the interaction of OsAHL1 with the promoter region of the target genes.

We analyzed the potential sequence signature of these promoters and the intron by MEME^15^. The alignment result revealed a degenerated 21 bp A/T rich sequence: WTWAWWWWTTTTTMAMWAANW (Fig. 10B). This implies that OsAHL1 could specifically bind to the A/T rich sequence region of promoters or introns, and hence regulate the expression of the downstream genes.

**Figure 12:**
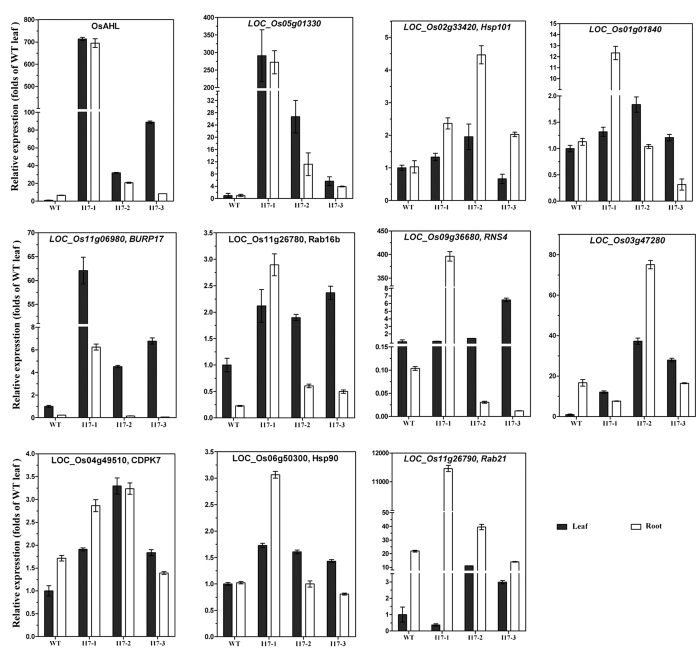
*OsAHL1* enhanced the expression of the target genes and other drought- related genes in rice. The relative expression of the target genes and other drought- related genes in the leaf and root of *OsAHL1* overexpression lines and WT plants under normal condition was quantified by qPCR. Error bars are the SD based on 3 replicates.

Furthermore, we detected the difference in the expression levels of the OsAHL1 target genes and some other drought tolerance related genes when comparing *OsAHL1* overexpression plant lines and the WT plants. Results indicated that most of the direct target genes of OsAHL1including *HSP101, OsCDPK7, OsRNS4, Rab16b, AP2-ERFBP like* (LOC_Os07g47790) and drought resistant related genes *HSP90, Rab21* were up-regulated in the *OsAHL1* overexpression plant lines (Fig. 12).

*HSP101*^16^,^17^ and *HSP90*^18^,^19^,^20^ play a crucial role in thermo stress tolerance and significantly increase the tolerance of abiotic stresses in plants. The dehydrin *Rab16b* and *Rab21* belong to LEA family. As a Ca^2+^ dependent protein kinase, *OsCDPK7* plays a key role in stress signal transduction and is a positive regulator involved in the tolerance to cold, salt and drought stress in rice^21^,^22^,^23^. *OsRNS4* is an S-like ribonuclease gene which regulates the photoresponse and ABA-induced growth inhibition in rice and its own expression is regulated by salt, PEG and ABA^24^,^25^. It may play an important role in reducing the degradation of Chlorophyll^25^. All of these genes were regulated by *OsAHL1* directly thus enhancing the drought avoidance and tolerance in rice plants.

## Discussion

### QTL mapping combined with gene expression microarray analysis is an effective method to identify complex trait related genes

There has been a lot of work on mapping of drought resistant traits and many drought resistance QTLs had since been identified; some of which have been verified and been applied efficiently in breeding practices^11^,^26^,^27^,^28^,^29^,^30^,^31^,^32^. However, there are relatively few reports on exploring drought resistant genes through the QTLs mapping. This is mainly because drought resistance of plants is a very complex trait and the secondary traits of drought resistance are not well understood. It is difficult to simplify drought resistant traits. Yue *et al.*^10^ had mapped the *AHL* gene in the QTL region related to rice root volume and canopy temperature on chromosome 11. This QTL was a large segment of sequences containing many genes. It is difficult to continue carrying out fine mapping using traditional QTL mapping and is almost impossible to identify specific genes. We combined a gene expression microarray with QTL mapping to explore functional genes and found the drought resistance gene *OsAHL1*. Follow-up experiments confirmed that overexpression of *OsAHL1* maintained rice root systems under drought stress and hence increased drought tolerance significantly. This approach was proven to be an effective way to explore functional genes inside the region of complicated trait QTLs.

### *OsAHL1* improved both drought tolerance and drought avoidance in rice plant

Luo and Zhang^33^ divided drought resistance into 4 types including drought tolerance, drought escape, drought avoidance and drought recovery. Yue *et al.*^10^ found in their study on rice root related QTL alleles that drought tolerance and drought avoidance could be controlled by different QTLs and were genetically separated by different mechanism. Our experiment results indicated that OsAHL1 not only affected rice root development, leaf water content, water loss rate of the leaf and traits associated with drought avoidance, but also affected drought tolerance related traits such as chlorophyll content, POD. This suggests that drought tolerance and drought avoidance can be genetically regulated by the same molecular pathway or through the same gene regulation. Therefore, the inheritance of drought tolerance and drought avoidance is closely correlated and interact with one another; they are not separated. This is a new aspect of OsAHL1 in abiotic stress resistance.

Vascular bundles are important tissues for perception and transduction of water stress signals in plants^34^. And *OsCDPK7* was known to be a positive regulator commonly involved in the tolerance to salt/drought stresses in rice^22^. Hear in our study, under drought stress the expression of the *OsCDPK7* gene controlled by *OsAHL1* was up-regulated in the vascular bundles in rice root and leaf. This up-regulation adjusts the processes of plant growth and development and activates downstream drought resistance genes^21^,^22^,^23^, which help organizations such as the rice root system to adapt to drought and further improve drought avoidance. *OsAHL1* could regulate the gene expression of RNS4, which in turn is beneficial to the stability of chlorophyll in the leaf under drought stress^25^. At the same time, *OsAHL1* directly activates the expression of drought tolerance related genes such as Rab16b, Rab21, HSP90 and HSP101 protein genes; thus enhancing the plant’s resistance to stress.

### Molecular mode of AHL1 gene function

Fujimoto *et al.*^13^ indentified an AHL gene in Arabidopsis and found it is localized in the active transcription region on the surface of the nucleolus. Its conserved functional domain DUF296 was first nominated as PPC domain (Plants and Prokaryotes Conserved domain). PPC/DUF296 domain is widely distributed in plants and prokaryotes, but as to the specific molecular function and action model there is still not clear explanation. Consistent with previous studies^13^,^35^,^36^,^37^,^38^, in our study, the AHL/AT-hook specifically targets gene promoters or A/T rich cis-elements of introns, and thereafter regulates downstream gene expression. In addition, the AT-hook and PPC domains could each guide proteins into the nucleus independently, but *OsAHL1* does not have distinct transcriptional activation activity. So, the molecular function model of *OsAHL1* is different from the general sense of transcription factors. For its molecular mode regulating downstream gene expression, Zhao *et al.*^38^ proposed the “DNA-AHL-TF complex model” based on their study on AHL genes in Arabidopsis. AHL family proteins formed AHL complex through homologous/heterologous combination. The complex uses its AT-hook motifs to anchor itself to AT-rich DNA regions and recruit either transcription factors or other non-AHL proteins in order to regulate plant growth and development. Such transcription factors interacting with AHL have been identified^38^. Correspondingly, we detected the homologous combination capability of the OsAHL protein (Fig. 9), and revealed for the first time that the OsAHL and DNA interacting *cis*-element region could be located either in the promoter region of target genes or within the gene sequence (intron) then activated the flanking gene expression, which enrich and validate this model to some extent.

The AtAHL22 gene controls flowering time by inhibiting FT gene expression^39^ and AtGIK inhibits ETT/ARF3 expression to regulate reproductive organ patterning and differentiation^40^. Here in our study, OsAHL1 activated the downstream gene expression to enhance increased drought resistance in rice, which is different from that of AtAHL22. Based on these experimental results and the “DNA-AHL-TF complex” model, it is presumed that different transcriptional factors bound by the AHL protein determine different pathways of regulating flanking genes. Therefore, the focus of our next research will be in depth analysis of the AHL interacting protein types and regulation pathways benefit to enhance DNA-AHL-TF complex models, which will help to reveal the role of its molecular model.

## Methods and Material

### Gene constructs and rice transformation

The native promoter [an upstream fragment (1,753 bp) starting from the base next to the start codon of *OsAHL1*] was amplified from genomic DNA of upland rice cv. IRAT109 and placed to pCAMBIA1300-EGFP to control GFP expression. Truncated and full-length cDNA of *OsAHL1*, without the termination codon, were placed respectively on pCAMBIA1300-EGFP for fusion expression under the control of the CaMV 35S promoter.

The full-length cDNA of *OsAHL1* was amplified from IRAT109 by RT-PCR and inserted into pCAMBIA1300 under the control of the CaMV 35S promoter for overexpression (I17). To make a dsRNAi construct of *OsAHL1* (RNAi) a 189 bp fragment of *OsAHL1* (nucleotides 1143–1331) was generated by PCR and was cloned into pCB2004B_2_ through *att*B/*att*P (BP) recombination cloning^41^. The *attB1* and *attB2* are the 2 sequences for the BP recombination reaction (Invitrogen). All of the constructs were transformed into the *japonica* rice cv. Zhonghua 11 by the *Agrobacterium*-mediated (stain EHA105) transformation method^42^.

### Site-directed mutagenesis of AHL1 (AHLmut) by PCR

A pair of reverse compliment long primers, AHL-mut F/R, were designed which contain the mutant sites (GRP to AAA). The wild type cDNA was used as template, part of the sequence from the OsAHL gene was amplified by the primer pairs of AHLaF/AHL-mutR and AHLaR/AHL-mutF respectively. Next, the mixture of products above was used as a template to amplify the full sequence using primer AHLaF/R. The majority of the products were mutant OsAHL.

### Plant growth, stress treatment, and measurement

Rice seeds of cultivar IRAT109 (*O. sativa* L. ssp. japonica) were germinated and grown in MS hydroponic culture medium for 18–20 days under normal growth conditions for rice. To measure the expression level of the *OsAHL1* gene under various stresses plants at the 4-leaf stage were removed from the water supply for the designated duration and were treated with abiotic stresses including drought, salt (150 mM NaCl), PEG6000 (20%), cold (exposing plants to 5 °C for the designated time), and phytohormones including ABA, gibberellic acid, jasmonic acid, salicylic acid, or ethephon.

Positive transgenic plants of *OsAHL1* T1 or T2 generation were selected by germinating seeds on MS medium containing 100 mg/L hygromycin. After germination, the positive seedlings were transplanted in hydroponic culture medium containing ABA, NaCl, and PEG6000. The drought stress at the panicle development stage was applied to plants grown in PVC tubes (1-m height, 20-cm diameter) by following the protocol of Yue *et al.*^10^. Genotypes of the plants were checked by PCR using hygromycin resistant gene-specific primers. Each stress test was repeated 3 or 4 times. RWC was progressively measured until the rolled leaf could not expand again after being supplied with water.

In order to calculate RWC of the leaf: the fresh weight (FW) of samples was measured, then fresh leaf segments were submerged in distilled water for 3 hours, the saturated weight (SW) was measured, and finally the leaves were oven dried at 120 °C for 24 h and the dry weight was recorded (DW). RWC was calculated according to:


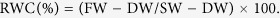


### Gene expression analysis

Total RNA was isolated from rice leaves using TRIzol reagent (Invitrogen). Real-time quantitative RT-PCR (qPCR) was performed on CFX96 Real-Time PCR System (Bio-Rad) using SYBR *Premix Ex Taq* (Takara). Rice *Actin1* gene was used as the endogenous control. The relative expression levels were determined as described previously^43^,^44^.

### Yeast two-hybrid assay

The yeast two-hybrid assay was performed using the GAL4 Two-Hybrid System (Stratagene). The entire or truncated coding region of OsAHL1 (or AHLmut) was amplified with primers Y2H. The PCR product was ligated into the pBD-GAL4 and pAD-GAL4-2.1 Cam phagemid vector according to the manufacturer’s instructions. The yeast strain YRG-2 (*Mata ura352 his3-200 ade2-101 lys2-801 trp1-901 leu2-3 112 gal4-542 gal80-538 LYS2::UAS*_*GAL1*_ *-TATA*_*GAL1*_*-HIS3 URA3::UAS*_*GAL4 17mers(x3)*_*-TATA*_*CYC1*_*-lacZ*) was transformed with both of the bait and prey plasmid according to the method described previously^45^.

### Yeast one-hybrid assay

The yeast one-hybrid was applied using the pGADT7 AD/pHIS2 system (Clontech). Genomic DNA was used as a template where the promoters and intron regions were amplified by PCR. All of the vectors were constructed by restriction enzyme digestion and ligation. The yeast strain Y187 (*Mata ura352 his3-200 ade2-101 trp1-901 leu2-3 112, galΔ, galΔ, MEL1, URA3:: GAL1*_*UAS*_*- GAL1*_*TATA*_ *-lacZ*) was transformed with both of the bait and prey plasmid according to the method described previously^45^.

### EMSA assay

Target probe was a PCR product obtained from the promoter region of the target gene. The desired DNA fragments was gel-purified and verified by restriction digestion. The probe was labeled by the Thermo Scientific Pierce Biotin 3′ End DNA Labeling Kit. And all of the following procedures were carried out according to the protocols described in Thermo Scientific Pierce Protein Ineraction Techinical Handbook.

### Microarray analysis

Two independent transgenic lines of *OsAHL1*overexpression and RNAi were used for microarray analysis. One month-old whole plants under normal/drought stress (20% PEG6000) condition were collected for microarray analysis. RNA sample preparation and hybridization were performed following the standard protocol of Affymetrix Gene Chip service (Shbiochip). Signal was normalized and estimated using the gcRMA package of Bioconductor^46^. Analysis of differential gene expression was performed using the LIMMA package^47^. The differential expression genes were filtered according to the following criteria: (i) assignment present in at least 2 chips; (ii) average log-transformed expression value by gcRMA larger than 3; and (iii) log-transformed changed fold more than 1 with expected value less than 0.05 by LIMMA^47^. The annotations of the probe sets, downloaded from Affymetrix and modified based on protein–protein BLAST analysis, were used for gene ontology analysis. Putative promoter sequences of the expression-level-changed genes were downloaded from the Gramene web site (http://cdna01.dna.affrc.go.jp/cDNA/). The cis-element enrichment analysis was carried by MEME (http://meme.nbcr.net/meme/cgi-bin/meme.cgi. Timothy L, 1994)^15^.

## Additional Information

**How to cite this article**: Zhou, L. *et al.* A novel gene *OsAHL1* improves both drought avoidance and drought tolerance in rice. *Sci. Rep.* **6**, 30264; doi: 10.1038/srep30264 (2016).

## References

[b1] Rabbani A. M. *et al.* Monitoring expression profiles of rice genes under cold, drought and high salinity stresses and abscisic acid application using cDNA microarray and RNA gel-blot analyses. Plant physiology 133, 1755–1767 (2003). 14645724 10.1104/pp.103.025742 PMC300730

[b2] Yamaguchi-Shinozaki K. & Shinozaki K. A novel cis-acting element in an Arabidopsis gene is involved in responsiveness to drought, low-temperature, or high-salt stress. Plant Cell 6, 251–264 (1994). 8148648 10.1105/tpc.6.2.251 PMC160431

[b3] Stockinger E. J., Gilmour S. J. & Thomashow M. F. *Arabidopsis thaliana* BBF1 encodes an AP2 domain-containing transcriptional activator that binds to the C-repeat/DRE, a cis-acting DNA regulatory element that stimulates transcription in response to low temperature and water deficit. Proc. Natl. Acad. Sci. USA 94, 1035–1040 (1997). 9023378 10.1073/pnas.94.3.1035 PMC19635

[b4] Ma S. & Bohnert H. J. Integration of Arabidopsis thaliana stress-related transcript profiles, promoter structures, and cell-specific expression. Genome Biol. 8, R49 (2007). 17408486 10.1186/gb-2007-8-4-r49 PMC1896000

[b5] Zhou L. *et al.* Genome-wide identification and analysis of drought-responsive microRNAs in *Oryza sativa*. J. Exp. Bot. 61, 4157–4168 (2010). 20729483 10.1093/jxb/erq237

[b6] Huang X. Y. *et al.* A previously unknown zinc finger protein, DST, regulates drought and salt tolerance in rice via stomatal aperture control. Genes & Development. 23, 1805–1817 (2009). 19651988 10.1101/gad.1812409 PMC2720257

[b7] Ge L. F. *et al.* Overexpression of the trehalose-6-phosphate phosphatase gene OsTPP1 confers stress tolerance in rice and results in the activation of stress responsive genes. Planta 228, 191–201 (2008). 18365248 10.1007/s00425-008-0729-x

[b8] Luo L. J. Breeding for water saving and drought resistance rice (WDR) in China. Journal of Experimental Botany. 61, 3509–3517 (2010). 20603281 10.1093/jxb/erq185

[b9] Pennisi E. The blue revolution, drop by drop, gene by gene. Science. 5873, 171–173 (2008). 18403686 10.1126/science.320.5873.171

[b10] Yue B. *et al.* Genetic basis of drought resistance at reproductive stage in rice: separation of drought tolerance from drought avoidance. Genetics 172, 1215–1228 (2006). 10.1534/genetics.105.045062 PMC1456219 16272419

[b11] Yue B. *et al.* Genetic analysis for drought resistance of rice at reproductive stage in field with different types of soil. Theor. Appl. Genet. 111, 1127–1136 (2005). 16075205 10.1007/s00122-005-0040-1

[b12] Kazuko Y. S. *et al.* Monitoring expression profiles of rice genes under cold, drought, and high-salinity stresses and abscisic acid application using cDNA microarray and RNA gel-blot analyses. Plant Physiology 133, 1755–1767 (2003). 14645724 10.1104/pp.103.025742 PMC300730

[b13] Fujimoto S. *et al.* Identification of a novel plant MAR DNA binding protein localized on chromosomal surfaces. Plant Mol. Biol. 56, 225–239 (2004). 15604740 10.1007/s11103-004-3249-5

[b14] Lin Y. *et al.* Crystal structure of pyrococcus horikoshii PPC protein at 1.60 A resolution. Proteins 67, 505–507 (2007). 17295322 10.1002/prot.21270

[b15] Timothy L. B. & Charles E. Fitting a mixture model by expectation maximization to discover motifs in biopolymers. Proceedings of the Second International Conference on Intelligent Systems for Molecular Biology, pp. 28–36, AAAI Press, Menlo Park, California (1994). 7584402

[b16] Chang C. *et al.* Transactivation of protein expression by rice HSP101 in plants and using Hsp101 as a selection marker for transformation. Plant Cell Physiol. 48, 1098–1107 (2007). 17597080 10.1093/pcp/pcm080

[b17] Wu T. *et al.* Interplay between heat shock proteins HSP101 and HSA32 prolongs heat acclimation memory posttranscriptionally in Arabidopsis. Plant Physiology 161, 2075–2084 (2013). 23439916 10.1104/pp.112.212589 PMC3613477

[b18] Ali M. *et al.* Crystal structure of an Hsp90-nucleotide-p23/Sba1 closed chaperone complex. Nature 440, 1013–1017 (2006). 16625188 10.1038/nature04716 PMC5703407

[b19] Liu D. D. *et al.* Enhanced thermotolerance of *E. coli* by expressed OsHsp90 from rice (Oryza sativa L.). Current microbiology 58, 129–133 (2009). 18946700 10.1007/s00284-008-9288-4

[b20] Lim S. D. *et al.* The rice RING finger E3 ligase, OsHCI1, drives nuclear export of multiple substrate proteins and its heterogeneous overexpression enhances acquired thermo tolerance. J. Exp. B. 64, 2899–2914 (2013). 10.1093/jxb/ert143 PMC3741691 23698632

[b21] Yusuke S. *et al.* Over-expression of single Ca^2+^-dependent protein kinase confers both cold and salt/drought tolerance on rice plants. The plant Journal 23, 319–327 (2000). 10929125 10.1046/j.1365-313x.2000.00787.x

[b22] Yusuke S. *et al.* A Ca^2+^-dependent protein kinase that endows rice plants with cold- and salt-stress tolerance functions in vascular bundles. Plant cell physiol. 42, 1228–1233 (2000). 10.1093/pcp/pce158 11726707

[b23] Huang G. T. *et al.* Signal transduction during cold, salt, and drought stresses in plants. Mol. Biol. Rep. 39, 969–987 (2012). 21573796 10.1007/s11033-011-0823-1

[b24] MacIntosh G. C. *et al.* RNase T2 genes from rice and the evolution of secretory ribonucleases in plants. Mol. Genet. Genomics 283, 381–396 (2010). 20182746 10.1007/s00438-010-0524-9

[b25] Zheng J. *et al.* Overexpression of an S-like ribonuclease gene, OsRNS4, confers enhanced tolerance to high salinity and hyposensitivity to phytochrome-mediated light signals in rice. Plant Science 214, 99–105 (2014). 24268167 10.1016/j.plantsci.2013.10.003

[b26] Yadav R. *et al.* Mapping genes controlling root morphology and root distribution in a doubled haploid population of rice. Theor. Appl. Genet. 94, 619–632 (1997).

[b27] Clark L. J., Aphale S. L. & Barraclough P. B. Screening the ability of rice roots to overcome the mechanical impedance of wax layers: importance of test conditions and measurement criteria. Plant and Soil 219, 187–196 (2000).

[b28] Lilley J. M. *et al.* Locating QTL for osmotic adjustment and dehydration tolerance in rice. J. Exp. Bot. 47, 1427–1436 (1996).

[b29] Tripathy J. N. *et al.* QTLs for cell-membrane stability mapped in rice (Oryza sativa L.) under drought stress. Theor. Appl. Genet. 100, 1197–1202 (2000).

[b30] Zou G. H. *et al.* Grain yield responses to moisture regimes in a rice population: association among traits and genetic markers. Theor. Appl. Genet. 112, 106–113 (2005). 16231161 10.1007/s00122-005-0111-3

[b31] Liu G. L. *et al.* Panicle water potential, a physiological trait to identify drought tolerance in rice. Journal of Integrative Plant biology 49, 1464–1469 (2007).

[b32] Zou G. H. *et al.* Screening for drought resistance of rice recombinant inbred populations in the field. Journal of Integrative Plant biology. 49, 1508–1516 (2007).

[b33] Luo L. J. & Zhang Q. F. The status and strategy on drought resistance of rice (Oryza sativa L.). Chinese J. Rice Sci. 15, 209–214 (2001).

[b34] Akira E. *et al.* Vascular system is a node of systemic stress responses. Plant Signaling & Behavior 3, 1138–1140 (2008). 19704460 10.4161/psb.3.12.7145 PMC2634481

[b35] Aravind L. & Landsman D. AT-hook motifs identified in a wide variety of DNA-binding proteins. Nucleic. Acids. Res. 26, 4413–4421 (1998). 9742243 10.1093/nar/26.19.4413 PMC147871

[b36] Strick R. & Laemmli U. K. SARs are cis DNA elements of chromosome dynamics: synthesis of a SAR repressor protein. Cell 83, 1137–1148 (1995). 8548801 10.1016/0092-8674(95)90140-x

[b37] Reeves R. & Nissen M. S. The A.T-DNA-binding domain of mammalian high mobility group I chromosomal proteins. A novel peptide motif for recognizing DNA structure. J. Biol. Chem. 265, 8573–8582 (1990). 1692833

[b38] Zhao J. F. *et al.* Arabidopsis thaliana AHL family modulates hypocotyl growth redundantly by interacting with each other via the PPC/DUF296 domain. PNAS. 10, E4688–E4697 (2013). 24218605 10.1073/pnas.1219277110 PMC3845178

[b39] Yun J. *et al.* The AT-hook motif-containing protein AHL22 regulates flowering initiation by modifying *FLOWERING LOCUS T* chromatin in Arabidopsis. Journal of Biological Chemistry 287, 15307–15316 (2012). 22442143 10.1074/jbc.M111.318477 PMC3346147

[b40] Ng K. H., Yu H.& Ito T. AGAMOUS Controls GIANT KILLER, a Multifunctional Chromatin Modifier in Reproductive Organ Patterning and Differentiation. PLOS Biology 7, e1000251 (2009). 19956801 10.1371/journal.pbio.1000251 PMC2774341

[b41] Lei Z. *et al.* High-throughput binary vectors for plant gene function analysis. J. Int. Plant Biol. 49, 556–567 (2007).

[b42] Hiei Y. *et al.* Efficient transformation of rice (Oryza sativa L.) mediated by Agrobacterium and sequence analysis of the boundaries of the T-DNA. Plant J. 6, 271–282 (1994). 7920717 10.1046/j.1365-313x.1994.6020271.x

[b43] Lefever S. *et al.* on behalf of the RDML consortium. RDML: structured language and reporting guidelines for real-time quantitative PCR data. Nucleic. Acids. Res. 37(7), 2065–2069 (2009). 19223324 10.1093/nar/gkp056 PMC2673419

[b44] Livak K. J. & Schmittgen T. D. Analysis of relative gene expression data using real-time quantitative PCR and the 2−∆∆CT method. METHODS 25, 402–408 (2001). 11846609 10.1006/meth.2001.1262

[b45] Gietz R. D., Schiestl R. H., Willems A. R. & Woods R. A. Studies on the transformation of intact yeast cells by the LiAc/SS-DNA/PEG procedure. Yeast 11, 355–360 (1995). 7785336 10.1002/yea.320110408

[b46] Gentleman R. C. *et al.* Bioconductor: Open software development for computational biology and bioinformatics. Genome Bio.l 5, R80 (2004). 10.1186/gb-2004-5-10-r80 PMC545600 15461798

[b47] Smyth G. K. Linear models and empirical Bayes methods for assessing differential expression in microarray experiments. Stat. Appl. Genet. Mol. Biol. 3, 1–25 (2004). 10.2202/1544-6115.1027 16646809

